# Esculentoside A Alleviates Intestinal Dysmotility in Ulcerative Colitis by Regulating H_2_S/CSE and NO/nNOS Systems

**DOI:** 10.1155/2022/7757833

**Published:** 2022-08-31

**Authors:** Ying Liu, Wenhua Wei, Shiwei Liang, Haicheng Fang, Jie Cao

**Affiliations:** ^1^Department of Gastroenterology, Guangxi Health Commission Key Laboratory of Glucose and Lipid Metabolism Disorders, The Second Affiliated Hospital of Guilin Medical University, Guilin, China; ^2^Department of Radiology, The Second Affiliated Hospital of Guilin Medical University, Guilin, China

## Abstract

**Background:**

Ulcerative colitis (UC) is a type of inflammatory bowel disease (IBD) that commonly affects the health of many individuals. Esculentoside A (EsA), a saponin extracted from the roots of *Phytolacca esculenta*, has antioxidative and anti-inflammatory effects against various diseases. Nonetheless, its role in UC is undetermined. Hence, in this study, we examined the therapeutic effects of EsA in UC.

**Methods:**

Primary intestinal neuronal cells (*in vitro*) were treated with lipopolysaccharide (LPS) to induce inflammatory injury. An *in vivo* UC rat model was created by the administration of dextran sulfate sodium (DSS) to rats, which were subsequently treated with different doses of EsA. The effects of EsA on intestinal motility, histological score, inflammatory response, hydrogen sulfide (H_2_S)/cystathionine *γ*-lyase (CSE) system, NO/neuronal nitric oxide synthase (nNOS) system, and LPS-induced primary intestinal neuronal cell viability loss, proliferation inhibition, and apoptosis were detected.

**Results:**

*In vitro*, EsA treatment increased the number of DSS-inhibited bowel movements and body weight, improved the histological score of colitis, and inhibited the inflammatory response by reducing IL-6 and TNF-*α* levels in rats. More importantly, EsA reduced the NO and H_2_S levels in serum and CSE, CBS, and nNOS expressions in the colon tissue. *In vivo*, EsA treatment eased the viability loss, proliferation inhibition, and apoptosis of LPS-stimulated primary intestinal neuronal cells, as well as inhibited the expressions of IL-6, TNF-*α*, CSE, CBS, and nNOS in cells.

**Conclusion:**

EsA improved intestinal motility and suppressed inflammatory response in DSS-induced UC, which may be mediated by H_2_S/CSE and NO/nNOS systems.

## 1. Introduction

As an inflammatory bowel disease (IBD), ulcerative colitis (UC) is usually triggered by a combination of genetic and environmental factors and is manifested by epithelial barrier damage, homeostasis disruption, and accumulation of inflammatory cells in the intestinal tract [1, 2]. The primary clinical features of UC include abdominal pain, rectal urgency, weight loss, and bloody diarrhea [1]. Although nonsteroidal anti-inflammatory drugs have been proven effective for UC, their adverse effects, including neoplasia and infection, cannot be neglected [3, 4]. Therefore, it is imperative to explore novel strategies for the treatment of UC.

The incidence of UC is closely associated with heredity, intestinal flora, immune disorders, eating allergies, and depression [5]. Most studies in the literature have focused on the immune pathogenesis of UC, including the role of abnormal immune responses and inflammatory factors [6].

Recently, abnormal intestinal motility associated with UC has attracted immense attention [7, 8]. Gastrointestinal motility contributes to the maintenance of the normal digestive function and is innervated by multiple nerves [7]. The Cajal stromal cells in the intestine act as pacemakers for gastrointestinal smooth muscle cells by generating slow waves of contraction, thereby controlling the rhythm, peristaltic direction, and speed of smooth muscle contraction [8]. The enteric nervous system (ENS) secretes excitatory neurotransmitters, such as acetylcholine, tachykinin, opioids, and 5-HT, and inhibitory neurotransmitters, such as vasoactive intestinal peptide and NO, that regulate intestinal motility. Either deficiency or excess of these neurotransmitters can affect smooth muscle contraction, causing gastrointestinal motility disorders [9]. Thus, the ENS may play a significant role in the mediation of UC.

Since the immense practical and social value of Chinese medicine is gradually emerging, the screening of applicable medical herbs for UC is of significance. Esculentoside A (EsA) is a traditional Chinese medicine derived from the roots of *Phytolacca esculenta* and contains highest content of saponins [10]. Several studies have demonstrated the antioxidative and anti-inflammatory effects of EsA in various experimental acute and chronic inflammatory and autoimmune diseases [10–13]. However, there are no reports on whether EsA can alleviate UC by regulating the ENS.

To this end, we explored the role of EsA in the treatment of dextran sulfate sodium (DSS)-induced UC and clarified the regulatory mechanism of the ENS via *in vitro* and *in vivo* experiments. These findings can provide theoretical and practical bases for the extensive application of EsA and other treatment strategies for UC using traditional Chinese medicine.

## 2. Materials and Methods

### 2.1. Animal Experiments

Thirty-two Sprague–Dawley (SD) rats (male, 5–6 weeks old) were obtained from Guilin Medical University animal center and housed in plastic cages. The rats were housed in a humidity of 60%–70% and controlled temperature of 22 ± 2°C and subjected to a 12 h light/dark cycle. Rats had free access to standard food and tap water. This study was approved by the ethics committee of Guilin Medical University and compliant with the ARRIVE guidelines.

After adaptation for a week, the rats were randomly divided into four groups: sham, model, EsA-10, and EsA-20 groups, with eight rats in each group. Rats in the model, EsA-10, and EsA-20 groups were administered 2% DSS (MP Biomedicals, CA, USA) via daily drinking water for eight days to induce UC. Furthermore, rats in the EsA-10 and EsA-20 groups were treated with 10 mg/kg and 20 mg/kg EsA, respectively (Shanghai Yuanye Biological Co., Ltd., China) via daily oral gavage for two weeks. The body weight and the number of bowel movements were measured per day to evaluate the disease severity. Finally, all rats were anesthetized via intraperitoneal injection of pentobarbital sodium (30 mg/kg, Shanghai Haoran Biological Technology Co., Ltd., Shanghai, China) and then sacrificed via cervical dislocation. The colon tissues were excised and the serum was collected for subsequent measurements.

### 2.2. Carbon Powder Propulsion Experiment

Rats were randomly selected from the four groups for the carbon powder propulsion experiment. In brief, the rats were given 10 mL/kg mixed solution of activated carbon (10%) and gum arabic (5%) by oral gavage. After 25 min, the rats were euthanized, and the gastrointestinal tract was dissected by laparotomy. The length of carbon powder propulsion in the intestine was then measured.

### 2.3. Histological Evaluation

Hematoxylin and eosin (H&E) staining was used to evaluate the histological alterations of colon tissues. In brief, after fixation with 4% paraformaldehyde, colon tissues were embedded in routine paraffin. After dewaxing and hydration, the sections were stained with H&E (C0105S, Beyotime Biotechnology, Shanghai, China) and observed under a light microscope (Nikon, Japan). Histological scores were independently allotted by two blinded researchers based on the following criteria: inflammatory cell infiltration (zero for occasional inflammatory cells in the lamina propria, one for increased inflammatory cells in the lamina propria, two for confluence of inflammatory cells extending into the submucosa, and three for transmural extension of the infiltrate) and tissue damage (zero for no mucosal damage, one for lymphoepithelial lesions, two for surface mucosal erosion or focal ulceration, and three for extensive mucosal damage and extension into deeper structures of the bowel wall) [14].

### 2.4. Immunohistochemistry

The sections were acquired similarly as in the H&E staining procedures. After dewaxing and hydration, the slides were exposed to citrate buffer. The slides were then pretreated at 80°C and incubated with 3% H_2_O_2_ to block endogenous peroxide. Thereafter, the slides were treated with anti-cystathionine *β*-synthase (CBS, ab140600, Abcam) antibody, anti-cystathionine *γ*-lyase (CSE, ab151769, Abcam) antibody, or antineuronal nitric oxide synthase (nNOS, ab1376, Abcam) antibody for 2 h at room temperature. After incubation with the relevant secondary antibody, the slides were mounted and analyzed using a light microscope (Nikon).

### 2.5. Separation of Primary Intestinal Neuronal Cells

The intestinal tissues were excised from healthy male SD rats and then cut into 5 cm segments on ice. After the intestinal contents were discarded, the muscular layer was dissected from the intestine and cut into pieces, followed by digestion at 37°C with 5% carbon dioxide and filtration with a 100 *μ*m mesh filter. Subsequently, the filtrate was collected and centrifuged at 4°C at 1000 rpm for 5 min. The cell precipitate after centrifugation was incubated in complete Dulbecco's modified eagle medium (DMEM) (Thermo, China), and primary intestinal neuronal cells were identified by the expression of neuron-specific enolase (NSE) using cell immunofluorescence.

### 2.6. Cell Treatments

Cell inflammation was induced by exposure to LPS (2.5 *μ*g/mL, Solarbio, Beijing, China). To analyze the impact of EsA on intestinal neuronal cell inflammation, different doses of EsA (10 and 20 *μ*M) were exposed to the LPS-induced intestinal neuronal cells.

### 2.7. Cell Immunofluorescence

The primary intermuscular nerve cells were identified by the NSE expression. In brief, at room temperature, cells were fixed in 4% formaldehyde solution for 12 min and then washed thrice with phosphate-buffered saline. Next, the cells were treated with 5% bovine serum albumin blocking buffer, followed by incubation with primary antibodies that target NSE (ab250275, Abcam; 1 : 200) and secondary antibodies at 4°C overnight and at 25°C for 1–2 h. Next, the cells were stained with 4′, 6-diamidino-2-phenylindole (DAPI, 28718-90-3, Sigma-Aldrich, MO, USA) for nuclear staining and examined under a fluorescence microscope.

### 2.8. Cell Viability Assays

In brief, the primary intestinal neuronal cells were seeded in 96-well plates and treated as described previously for 1, 2, or 3 days. Next, they were incubated with CCK-8 (C0038, Beyotime Biotechnology) for 2 h, and the OD450 value was obtained using a microplate reader.

### 2.9. 5-Ethynyl-2′-Deoxyuridine Assay

The cell proliferation assay was performed using a 5-Ethynyl-2′-deoxyuridine (EdU) kit (E10187, Invitrogen, CA, USA). In brief, the primary intestinal neuronal cells were treated as described previously for 48 h and labeled with EdU for 2 h. Then, the cells were fixed in 4% paraformaldehyde followed by sequential staining with Apollo (R11076, RioBio Corporation, Shanghai, China) and DAPI and were observed under an inverted microscope (Olympus).

### 2.10. Cell Apoptosis Assay

The cell apoptosis assay was carried out using an Annexin V-fluorescein isothiocyanate (FITC)/propidium iodide (PI) kit (APOAF, Thermo Fisher Scientific, MA, USA). The primary intestinal neuronal cells were treated as described previously for 24 h and sequentially treated with Annexin V-FITC and PI. A flow cytometer (BD Bioscience, NJ, USA) was used to evaluate the results of the cell apoptosis assay.

### 2.11. Quantitative Reverse Transcription-Polymerase Chain Reaction

Total RNA was isolated from the treated cells and tissues using Invitrogen™ TRIzol™ reagent (R0016, Beyotime Biotechnology). Then, QuantiTect Reverse Transcription Kit (205311, Qiagen, MD, USA) was used to obtain complementary DNA, and quantitative polymerase chain reaction was performed based on the guidance of the SYBR Premix Ex Taq TM II (RR420, Takara Biotechnology, Beijing, China). The primer sequences are displayed in Table 1. The relative expressions of CSE, CBS, and nNOS were detected, and GAPDH was set as the internal control. Data were calculated by the 2^−∆∆Ct^ method [15].

### 2.12. Western Blotting

Total proteins were obtained from the cells or tissues on ice using a lysis buffer (P0013, Beyotime Biotechnology), separated using sodium dodecyl sulfate-polyacrylamide gel electrophoresis (SDS-PAGE), and then transferred to a polyvinylidene difluoride membrane. After blocking the membrane and treating with mammalian target of CSE (ab151769), CBS (ab140600), nNOS (ab1376), or GAPDH (ab8245, 1 : 1000, Abcam) antibodies for 3 h at room temperature, the membrane was incubated with the secondary antibody (1 : 3000, Abcam). Finally, the protein expression was evaluated using enhanced chemiluminescence (ECL, Millipore, USA).

### 2.13. Detection of Inflammatory Factor Levels

The expression levels of tumor necrosis factor-*α* (TNF-*α*) and interleukin 6 (IL-6) were detected in the serum and cell samples using an enzyme-linked immunosorbent assay kit (ELISA) (purchased from Solarbio Life Science Company; SEKR-0005, SEKR-0009, Beijing, China) as per the manufacturer's instructions. The relative contents of H_2_S and NO in the serum and cells were tested using microplate assay kits (BC2050, BC1475, Solarbio Life Science Company).

### 2.14. Statistical Analysis

Quantitative data were analyzed for statistical significance using the SPSS 20.0 software (IBM Corp., Armonk, NY, USA). One-way ANOVA followed by Tukey's post hoc test was utilized to compare the differences among various groups. *p* < 0.05 was set as the threshold of statistical significance.

## 3. Results

### 3.1. EsA Ameliorated DSS-Induced UC in Rats

**Figure 1:**
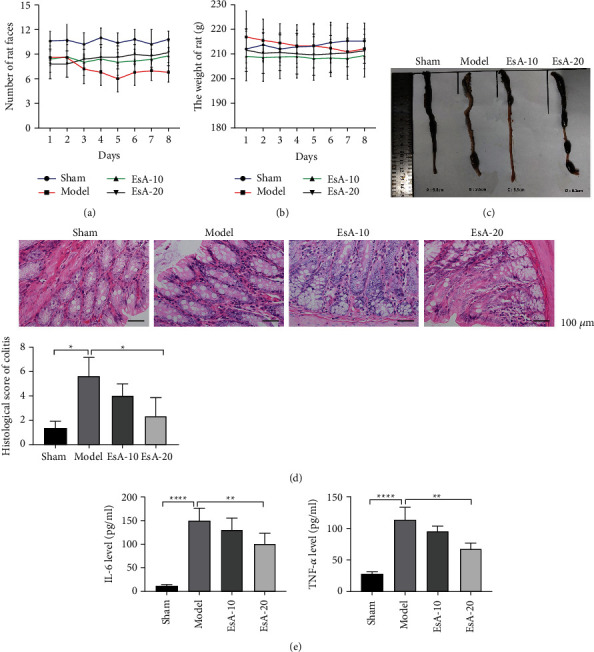
The therapeutic effects of Esculentoside A on dextran sulfate sodium (DSS)-induced ulcerative colitis (UC). (a) The number of rat bowel movements in the sham, model, Esculentoside A (EsA)-10, and EsA-20 groups. (b) The body weight of rats in the sham, model, EsA-10, and EsA-20 groups. (c) The length of carbon powder propulsion in the sham, model, EsA-10, and EsA-20 groups in the carbon powder propulsion experiment. (d) Hematoxylin and eosin staining in the sham, model, EsA-10, and EsA-20 groups. (e) The levels of TNF-*α* and IL-6 in the serum samples of the sham, model, EsA-10, and EsA-20 groups. ^*∗*^*p* < 0.05, ^*∗*^*p* < 0.01, and ^*∗∗∗∗*^*p* < 0.0001 vs. model group by one-way ANOVA followed by Tukey's post hoc test (*n* = 3).

To evaluate the effects of EsA on UC, different doses of EsA were administered to the DSS-induced UC rat model. DSS treatment decreased the number of bowels (Figure 1(a)), while slightly lowered the body weight (Figure 1(b)). The carbon powder propulsion experiment revealed that the length of carbon powder propulsion was shorter in the model group than in the sham group (2.6 cm vs. 9.8 cm, Figure 1(c)). Meanwhile, after DSS treatment, H&E staining assay demonstrated that colonic tissue injuries and histological score of UC were remarkably increased (Figure 1(d)). Moreover, the concentrations of IL-6 and TNF-*α* in the serum samples of the DSS group were elevated (Figure 1(e)). Taken together, these results suggested that a DSS-induced UC rat model was successfully established. Furthermore, EsA treatment increased the number of bowel movements (Figure 1(a)), body weight (Figure 1(b)), and the length of carbon powder propulsion (EsA-10 and EsA-20 groups vs. model group, 3.5 cm and 6.1 cm vs. 2.6 cm, Figure 1(c)). In addition, EsA improved tissue injuries, reduced the histological score of UC (Figure 1(d)), and inhibited TNF-*α* and IL-6 levels in the serum samples (Figure 1(e)). Notably, a high dose of EsA (20 mg/kg) exhibited a superior therapeutic effect compared with the low dose of EsA (10 mg/kg) for DSS-induced UC.

### 3.2. Effects of EsA on H_2_S/CSE and NO/nNOS Systems in a DSS-Induced UC Rat Model

**Figure 2:**
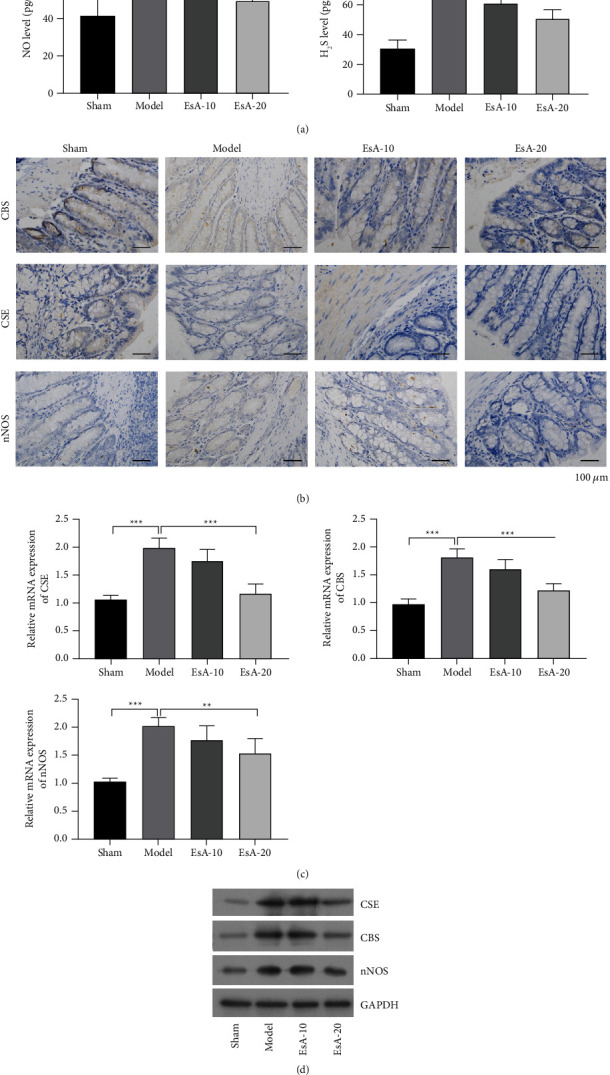
Effects of Esculentoside A on hydrogen sulfide/cystathionine *γ*-lyase and NO/neuronal nitric oxide synthase systems in the dextran sulfate sodium (DSS)-induced ulcerative colitis (UC) rat model. (a) The contents of hydrogen sulfide (H2S) and NO in serum samples of the sham, model, Esculentoside A (EsA)-10, and EsA-20 groups. (b) The expression of cystathionine *β*-synthase (CBS), cystathionine *γ*-lyase (CSE), and neuronal nitric oxide synthase (nNOS) in the sham, model, EsA-10, and EsA-20 groups by immunohistochemistry. (c, d) The mRNA and protein levels of CSE, CBS, and nNOS in the sham, model, EsA-10, and EsA-20 groups by quantitative reverse transcription polymerase chain reaction and western blotting. ^*∗*^*p* < 0.05, ^*∗*^*p* < 0.01, and ^*∗∗∗*^*p* < 0.001 vs. model by one-way ANOVA followed by Tukey's post hoc test (*n* = 3).

For understanding the molecular mechanisms underlying the alleviation of DSS-induced UC by EsA, we analyzed the role of H_2_S/CSE and NO/nNOS systems. Compared with the sham group, the model group had elevated concentrations of NO and H_2_S, which were in turn inhibited by EsA, especially in the EsA-20 group (*p* < 0.05, Figure 2(a)). In addition, the mRNA and protein levels of CSE, CBS, and nNOS were remarkably increased in the model group than in the sham group as revealed by the qPCR, western blotting, and IHC assays. However, EsA partly reversed their expression, especially in the EsA-20 group (*p* < 0.05, Figures 2(b)–2(d)).

### 3.3. Effects of EsA on LPS-Induced Intestinal Neuronal Cells

**Figure 3:**
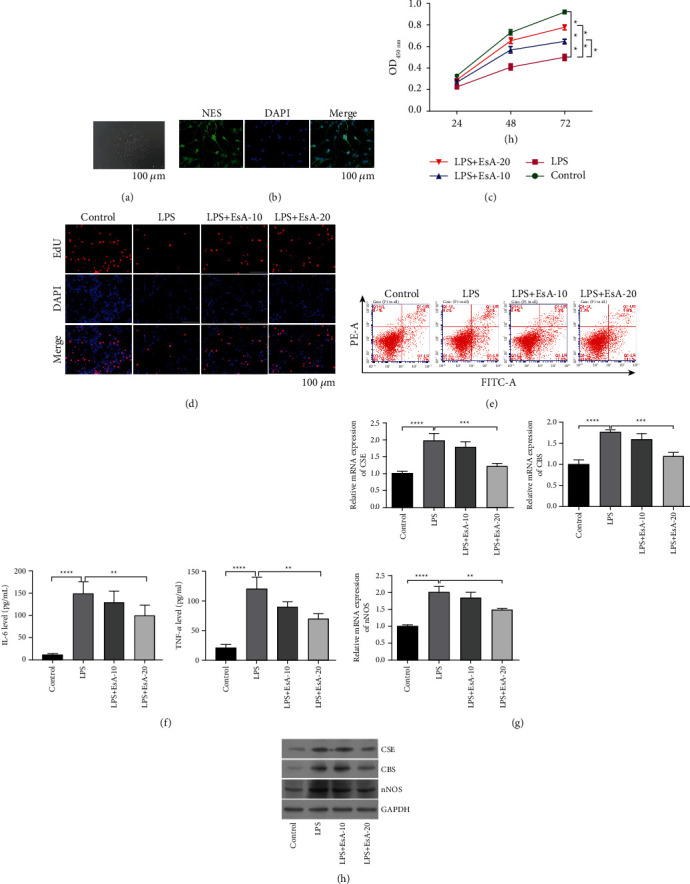
Effects of Esculentoside A on LPS-induced intestinal neuronal cells. (a) The cell morphology of primary intestinal neuronal cells. (b) The expression of neuron-specific enolase (NSE) in primary intestinal neuronal cells by cell immunofluorescence. (c–e) Cell viability, proliferation, and apoptosis of primary intestinal neuronal cells in control, lipopolysaccharide (LPS), Esculentoside A (EsA)-10, and EsA-20 groups assessed using the CCK-8 assay, 5-Ethynyl-2′-deoxyuridine (EdU) assay, and flow cytometry. (f) The contents of interleukin 6 (IL-6) and tumor necrosis factor-*α* (TNF-*α*) in the primary intestinal neuronal cells of control, LPS, EsA-10, and EsA-20 groups using enzyme-linked immunosorbent assay. (g) The mRNA and protein levels of cystathionine *γ*-lyase, cystathionine *β*-synthase, and neuronal nitric oxide synthase in primary intestinal neuronal cells of the control, LPS, EsA-10, and EsA-20 groups using quantitative reverse transcription polymerase chain reaction and western blotting. ^*∗*^*p* < 0.05, ^*∗*^*p* < 0.01, ^*∗∗∗*^*p* < 0.001, and ^*∗∗∗∗*^*p* < 0.0001 vs. LPS by one-way ANOVA followed by Tukey's post hoc test (*n* = 3).

In addition to the *in vivo* experiments, the *in vitro* effects of EsA on UC were investigated. We isolated primary intestinal neuronal cells from healthy SD rats. The primary intestinal neuronal cells tended to have a spindle cell pattern (Figure 3(a)) and exhibited a positive expression of NSE (Figure 3(b)), thereby suggesting that we had successfully isolated primary intestinal neuronal cells. LPS was used to inflict inflammatory injury in intestinal neuronal cells, which were compared with the control group. LPS significantly suppressed cell viability in a time-dependent manner (*p* < 0.001, Figure 3(c)) and decreased cell proliferation (Figure 3(d)). Meanwhile, especially high dose of EsA, significantly promoted cell viability in a time-dependent manner (*p* < 0.05, Figure 3(c)) and increased cell proliferation (Figure 3(d)) in LPS-induced intestinal neuronal cells. The cell apoptosis assay further revealed that the number of apoptotic cells had increased after LPS treatment, which was reversed by EsA in primary intestinal neuronal cells (Figure 3(e)). In addition, LPS significantly enhanced the expressions of TNF-*α* and IL-6 in cells, an effect that was partly reversed by EsA (*p* < 0.05, Figure 3(f)). Furthermore, the qPCR and western blotting results found that the expressions of CSE, CBS, and nNOS were upregulated in the LPS group compared with the control group. However, EsA reversed their expression induced by LPS (Figures 3(g)–3(h)).

## 4. Discussion

Abnormal intestinal motility is usually accompanied by the occurrence and development of UC [16]. A previous investigation demonstrated that abnormal intestinal motility in UC was jointly mediated by the central nervous system, ENS, and gastrointestinal smooth muscles [17]. Currently, several treatment options are employed for UC, including surgery, interventional therapy, nanomedicine, and Chinese traditional medicine [18]. EsA, a commonly used Chinese traditional medicine, has been proven to inhibit inflammatory response. However, little is known about its potential role in UC-induced abnormal intestinal motility. In this study, we established a DSS-induced UC mice model and found that EsA not only improved histopathological injury and intestinal motility but also inhibited the production of TNF-*α* and IL-6 in DSS-induced UC rats. Furthermore, this study also revealed that EsA reduced the expression of NO, H_2_S, CSE, CBS, and nNOS. This indicates the relationship of H_2_S/CSE and NO/nNOS systems with the therapeutic effect of EsA in DSS-induced UC rats. Consistently, EsA reversed cell inflammatory injury as well as the expression of NO, H_2_S, CSE, CBS, and nNOS induced by LPS in primary intestinal neuronal cells (*in vitro*).

The smooth muscle activity is majorly implicated in the abnormal intestinal motility induced by UC and is mainly regulated by various factors, including hormones, neurotransmitters, and inflammatory factors [7, 19]. TNF-*α* and IL-6 are important cytokines that play a key role in the pathogenesis of inflammatory diseases [20]. Previous studies have suggested the presence of high levels of TNF-*α* and IL-6 in the UC intestinal mucosa and that mucosal lesions further aggravate the release of inflammatory mediators, thereby influencing the activity of smooth muscle and altering intestinal motility [6, 21, 22]. Our results showed that EsA increased the number of bowel movements, body weight, and length of carbon powder propulsion and improved tissue injuries as well as inhibited the contents of TNF-*α* and IL-6 in the serum samples of DSS-induced UC rats. Similarly, the *in vitro* experiments revealed that EsA promoted cell proliferation, induced cell apoptosis, and reduced the expression of TNF-*α* and IL-6 in LPS-induced intestinal neuronal cells. Taken together, these data suggest that EsA exerted anti-inflammatory effects in UC.

Furthermore, we investigated the possible mechanism of action of EsA in the treatment of UC. NO and nNOS were found to be overexpressed in both colon tissues and LPS-induced intestinal neuronal cells, and EsA reversed the expression of NO and nNOS induced by DSS or LPS. A previous study reported the overexpression of nNOS and overproduction of NO in patients with IBD [23, 24]. Inflammatory cytokines such as TNF-*α* and IL-6 stimulate the upregulation of nNOS in inflammatory cells and thereby cause overproduction of NO [24, 25], which may disrupt the intestinal mucosal barrier and inhibit intestinal motility [26]. In addition, we found that the expressions of H_2_S, CSE, and CBS were obviously increased in the colon tissues and LPS-induced intestinal neuronal cells. In contrast, their expressions were inhibited by EsA treatment. H_2_S, synthesized from L-cysteine by either of the two cytosolic enzymes (CBS and CSE), is considered as a pro-inflammatory factor in several inflammatory diseases, including arthritis and pancreatitis [27, 28]. Notably, H_2_S is involved in relaxing gastrointestinal smooth muscles [29]. Considering these findings, we speculated that the anti-inflammatory effects of EsA on UC might be related to H_2_S/CSE and NO/nNOS systems.

## 5. Conclusion

In conclusion, this study unveiled the ability of EsA to ameliorate the inflammatory response and improve the intestinal motility in both DSS-induced UC rats and LPS-induced intestinal neuronal cells. Moreover, the study identified that H_2_S/CSE and NO/nNOS systems might be partly responsible for the therapeutic effects of EsA on UC.

## Figures and Tables

**Table 1:** Specific primers for quantitative reverse transcription polymerase chain reaction assay.

Genes	Primer sequence
*CSE*	Sense primer: 5′-AGAGGAGCTCTTGGAGACGACAT-3′
	Antisense primer: 5′-ACTCCTGCTTGTTAATT CCGACC-3′
*CBS*	Sense primer: 5′-AATGGTGACGCTTGGGAA-3′
	Antisense primer: 5′-TGAGGCGGATCTGTTTGA-3′
*nNOS*	Sense primer: 5′-CAGCCCAATGTCATTTCTGTT-3′
	Antisense primer: 5′-GATCACGGGCGGCTTACT-3′
*GAPDH*	Sense primer: 5′-GTGGATCAGCAAGCAGGAGT-3′
	Antisense primer: 5′-AAAGCCATGCCAATCTCATC-3′

## Data Availability

The datasets used and/or analyzed in the current study are available from the corresponding author on reasonable request.
